# COVID-19 Vaccine Information Exposure: The Effect of Online Authority vs. Non-Authority Sources on Beliefs, Emotions and Information Engagement Behaviors

**DOI:** 10.3390/vaccines12101096

**Published:** 2024-09-26

**Authors:** Xiaowen Xu, Carolyn A. Lin

**Affiliations:** 1 Department of Strategic Communication, Organizational Communication & Leadership, College of Communication, Butler University, Indianapolis, IN 46208, USA; xxu4@butler.edu; 2 Department of Communication, University of Connecticut, Storrs, CT 06269-1259, USA

**Keywords:** COVID-19 vaccines, Stimulus–Organism–Response (S-O-R) framework, Health Belief Model, vaccine information engagement, vaccination beliefs, perceived vaccination benefits vs. barriers, emotion toward vaccination, hope vs. fear, authority vs. non-authority COVID-19 information sources

## Abstract

**Background/Objectives:** Limited research has examined the theoretical linkages between exposure to COVID-19 vaccine information sources, vaccination-related beliefs, vaccination-induced emotions, and vaccine information engagement. **Methods:** An online survey was conducted with a national sample of adults (N = 630) residing in the U.S. to test these relationships, guided by the Stimulus–Organism–Response (S-O-R) framework and the Health Belief Model. **Results:** Study findings showed that exposure to online authority vaccine information sources was positively related to vaccination-benefit beliefs and negatively related to vaccination-barrier beliefs, in addition to hopeful feelings connected to vaccination. Exposure to non-authority sources was positively associated with vaccination-barrier beliefs, hopeful and fearful feelings connected to vaccination, and vaccine information engagement. While vaccination-benefit beliefs and vaccination-barrier beliefs were negatively and positively linked to vaccine information engagement, respectively, these beliefs were each positively connected to hopeful feelings and fearful feelings toward vaccination in that order. Both hopeful and fearful feelings toward vaccination also emerged as positive correlates of vaccine information engagement. **Conclusions:** This study contributes to our understanding of how cognitive appraisals of and affective responses to risk information disseminated by different types of sources may be related to risk information engagement behavior in a public health crisis. Results bring evidence-based insights to both researchers and health professionals to better equip them to counter vaccine misinformation and reduce vaccination barriers.

## 1. Introduction

During the rollout of COVID-19 vaccines, information disseminated from “authority sources” such as governments and medical professionals with scientific facts about the vaccines, was widely available to the public [1]. Not to be outdone, false and misleading information about the vaccines had also been prevalent in online media [2,3] and impacted the opinions of anti-vaccine groups to help shape their narrative against vaccines [4]. Such misinformation might have also negatively affected patients with chronic diseases who were undergoing immunosuppressive treatment, by fueling them with uncertainty and hesitancy toward COVID-19 vaccines [5,6]. Past studies have shown that an individual’s use of risk information sources was associated with pandemic knowledge [1], vaccination beliefs [7], and preventive behaviors (e.g., receiving a vaccine) [8,9,10]. Yet, the linkages between exposure to risk information sources and information-related behaviors have not been fully examined [11].

Information engagement as an information-related behavior in a risk communication context refers to behaviors practiced by individuals such as sharing and interacting with the risk information to manage emotions and cope with risks [12,13]. Past research has indicated that when individuals sought or consumed different types of information from various sources, they also had the means and ability to shape the information environment via their own information engagement behaviors [14,15]. In order to better understand the factors that contribute to the dissemination of vaccination information, it is important to consider how individuals engage with the information they have received [16] in relation to their exposure to different media sources.

Past research has shown that exposure to social media was shown to be related to engagement with COVID-19 information on these digital venues [17,18]. Yet, empirical research that examines audience engagement with COVID-19-related information shared across on different information source types has been lacking. Similarly, studies addressing how risk information sources may elicit cognitive and affective responses to a health threat such as COVID-19 and how these responses may drive further risk information engagement remains scarce. Therefore, empirical data are still in need to delineate the psychological process behind this information-related behavior. Furthermore, the existing literature on information engagement mostly focused on information about the COVID-19 pandemic instead of COVID-19 vaccines. For this reason, little is known about the psychological and behavioral implications of exposure to vaccine-specific information sources.

The current study aims to address the literature gap that could connect cognitive and affective responses toward the vaccines and the subsequent risk information engagement behavior (or the lack of it), after individuals have been exposed to different types of risk- information sources about the vaccines. By integrating the cognitive appraisal constructs from the Health Belief Model [19] with the Stimulus–Organism–Response (S-O-R) framework, this research explored the cognitive and affective reactions (O) to different COVID-19 vaccine information sources (S)—as well as how such dynamics acted as mediators for the effects of information sources on vaccine information engagement (R)—during the peak of the pandemic outbreak. This study contributed to the literature by offering a conceptual framework that links exposure to online authority vs. non-authority information sources to key cognitive and affective factors to explain behavioral engagement with vaccine information.

### 1.1. Vaccine-Related Information Engagement

Information engagement is characterized as behavioral manifestations with a message including interaction with the information (e.g., expressing thoughts and opinions such as exhibiting approval, disapproval, likes, dislikes, raising questions and comments on the subject), as well as interaction with others in association with the substance of the shared information (e.g., sharing and discussing the information with others) [20,21]. Through individual interaction with information in a social scenario, information engagement not only actively contributes to the information flow by spreading, relaying, and discussing information [14,15], but also constitutes an active coping strategy against the risk that one encounters [13,22]. Despite such importance, this particular “information action” and “coping function” performed via information engagement behavior is not yet well understood and warrants a more thorough theoretical and empirical examination.

As various media sources provide materials for and/or encourage information engagement behavior from the audiences [23,24], exposure to these sources should not be ignored when examining the nomological network of engagement with information. Existing research on information source exposure and information engagement in a pandemic situation is limited in scope. For example, exposure to information sources such as social media was shown to be related to engagement with COVID-19 information on these venues [17,18]. Yet, no evidence has been reported for other types of information sources.

In addition, the existing literature mostly focused on engagement with the information about the COVID-19 pandemic, instead of COVID-19 vaccines, leaving a gap in our knowledge about vaccine-specific information associated with different types of information sources. Relatedly, no research hitherto has examined the mechanism of how exposure to vaccine information sources may be linked to information engagement behaviors. In other words, we have no knowledge of what cognitive and affective responses may be elicited by exposure to different information outlets, and how such responses might have facilitated further risk information engagement during the peak of the pandemic. The current study aims to answer these questions by applying the Stimulus–Organism–Response (S-O-R) framework, as discussed in the ensuing section below.

### 1.2. Stimulus–Organism–Response (S-O-R) Paradigm

The Stimulus–Organism–Response (S-O-R) framework [25] conceptualizes the relationship between environmental factors and individuals’ internal states and behaviors as a sequence of cognitive and affective events. The framework posits that environmental sensory stimuli can elicit internal reactions (organisms) within individuals, which subsequently influence their approach or avoidance behaviors (responses) toward that environment. “Organism” is characterized by three sub-dimensions [25]: pleasure, arousal, and dominance. In particular, “pleasure–displeasure” pertains to the level of enjoyment and satisfaction—and “arousal–nonarousal” denotes the general state of physical and mental alertness—both of which represent emotional reactions. On the other hand, “dominance–submissiveness” (D) reflects feelings of control or lack of control in given situations and surroundings, which is connected to cognitive judgments [26].

Existing research has adopted the S-O-R framework to examine behaviors such as information avoidance [27,28] and purchasing behavior [29] in the context of the COVID-19 pandemic. However, this framework has not seen much use in information engagement research in the same research context. When adopting this framework, the constructs under study can be conceptualized as follows: (1) “information engagement” can be considered as a behavioral response (R) toward exposure to different sources for COVID-19 vaccine information; (2) “information sources” can be designated as typical stimuli (S) in the information environment; and (3) the cognitive and affective factors (O) can be seen as “information journey” variables that connect the process starting from information exposure and ending with information engagement. The discussion below will introduce each of these variables and hypothesize their conceptual linkages, as guided by the aforementioned S-O-R model.

### 1.3. Information Sources and Vaccination Beliefs

Research has shown that medical professionals and official sources such as government agencies (i.e., Center for Disease Control) were major COVID-19 information sources used by individuals, as these were regarded as “authority” sources [1]. A previous survey with populations in six different countries (e.g., U.S., Argentina, South Korea) reported that residents of these countries rated their national governments highly on disseminating trustworthy COVID-19 information [2]. Additional multi-country studies have also documented how the public had depended on social media, search engines, online video sites, and messaging applications to obtain COVID-19 and vaccine information, e.g., [2,30].

According to the Health Belief Model [19], when individuals encounter a health risk, they may assess whether they are subject to the risk and the level of that risk and then proceed to appraise the benefits and barriers associated with adopting the protection action suggested by the relevant information sources. While perceived barriers refer to perceived obstacles to enacting a protective health behavior, perceived benefits indicate the perceptions of effectiveness of a protective health action in addressing the health threat [19]. Hence, vaccination benefits vs. barriers each reflect an adaptive vs. a maladaptive approach toward the pandemic as a health threat [31]. Perceived benefits and barriers also align with the dimension of dominance–submissiveness of the “organism” concept in the S-O-R framework, as these two perceptions reflect internal cognitive reactions toward the vaccine-relevant information.

An abundance of research has studied the connection between media use and (1) pandemic knowledge and beliefs [7] and (2) pandemic prevention behavior [8,10]. Yet, research that examined the relationships between information sources and health beliefs about vaccines remains lacking. For instance, Zhao and Wu [32] reported that exposure to various online and offline news media (e.g., print, TV, website) positively predicted both perceived efficacy and perceived threat of vaccines. While social media use was found to be correlated with COVID-19 misinformation exposure [33,34], earlier work discovered that social media use (Twitter and Facebook) as sources of health information was associated with higher likelihood of influenza vaccination [35]. More particularly, online non-authority sources, especially those on social media are deemed as a hotbed for COVID-19-related misinformation [3] that casts doubt on or denies the effectiveness and safety of COVID-19 vaccines [36]. Even so, the literature has yet to address the specific influence of online non-authority media on perceived vaccination effectiveness and barriers.

In the current study context, online non-authority sources include those online venues (e.g., websites, social media) that share COVID-19 vaccine information without sourcing validated science-based evidence or interviewing credentialed medical experts, scientists or government health agencies. These online information sources that make up the Infodemic universe can include low-credibility tabloids, anti-vaccine groups, conspiracy theory believers, and the like, in addition to a wide variety of news, media and information websites [34]. Based on the limited relevant research, it is assumed that exposure to these online non-authority sources that share COVID-19 vaccine information may be negatively linked to perceived vaccination benefits and positively associated with perceived vaccination barriers. The hypotheses below are proposed to verify these assumptions.

**Hypothesis 1a–b (H1a–b).**  *Exposure to COVID-19 vaccine information disseminated by online non-authority sources will be (a) positively related to perceived vaccination barriers and (b) negatively related to perceived vaccination benefits.*

Public health officials and governments are ultimately responsible for providing scientific evidence and guidelines of disease prevention techniques against the pandemic [37] to debunk misinformation about COVID-19 vaccines [38]. Prior work reported that audience use and trust of authority sources (i.e., scientific organizations, medical professionals, local, state and national governments, and health agencies) resulted in significantly better knowledge and fewer misbeliefs about the COVID-19 pandemic [1,39], as well as a greater intention to receive vaccination [8,40,41].

Zimand-Sheiner et al. [42] also found that trust in governmental organizations positively mediated the effect of exposure to vaccine information on attitudes toward vaccination. However, empirical studies assessing the linkage between exposure to online authority sources and health beliefs about vaccines are non-existent. To fill this empirical gap, the current study focuses on the online presence of sources, such as government officials (e.g., the U.S. Surgeon General), public health officials (e.g., Dr. Anthony Fauci), medical correspondents (e.g., Dr. Sanja Gupta), medical professionals (e.g., Dr. Leena Wen) or research scientists (e.g., Drs. Katalin Karikó and Drew Weissman who developed the mRNA vaccines), that appear in various news, information, and media online including social media outlets.

As the literature has suggested that online authority sources that disseminate scientific facts about vaccines is related to an individual’s confidence in vaccination, e.g., [1,39], it is anticipated that exposure to these sources will be related to greater perceived vaccination benefits and lower perceived vaccination barriers. The following hypotheses are posited to test these assertions.

**Hypothesis 2a–b (H2a–b).**  *Exposure to COVID-19 vaccine information disseminated by online authority sources will be (a) negatively related to perceived vaccination barriers and (b) positively related to perceived vaccination benefits.*

### 1.4. Information Sources, Fear, and Hope

Aside from cognitive beliefs, affective reactions stand for another type of “organism” in response to the stimulus in the S-O-R framework. The construct of fear is considered as an “organism” of displeasure and arousal [43]. Fear for the COVID-19 vaccines—due to concerns for safety, side effects, and the rapid development of the vaccines—was identified as an affective barrier to vaccination [44,45]. The emotion of hope arises, when a stimulus offers relief, or a better outcome (to obtain a reward or avoid punishment) than the current, less favorable situation [43]. Smith and Lazarus [46] conceptualized hope as originating from an adaptive function of sustaining commitment and coping with the expectation of future success or amelioration of harmful conditions. Both fear and hope are prevalent emotions in the context of health crises, as noted in past research [47,48].

Lee et al. [49] reported that attention to news on online media (e.g., social media, blogs, Internet news portals, podcasts) increased perceived information load, which further predicted such negative emotions as stress and anxiety. Other studies similarly verified negative emotional implications of online media use for pandemic information, including anxiety, depression, and reduced emotional well-being [50,51]. Still, how different information sources may be related to discrete emotions toward the vaccines is largely unknown.

Logically, consumption of science-based information from online authority sources may strengthen individuals’ confidence in the vaccines, thus boosting hope—and reducing fear—toward the vaccines. By contrast, hope for the vaccine may be dampened—and fear heightened—after exposure to a large amount of misinformation that usually denies the effectiveness of vaccines seen in numerous online non-authority media [52]. To explore these yet-to-be verified relations between the usage of vaccine information sources and emotional responses, a set of hypotheses are proposed below.

**Hypothesis 3a–b (H3a–b).**  *Exposure to COVID-19 vaccine information disseminated by online non-authority sources will be (a) positively related to fear (connected to vaccination) and (b) negatively related to hope (connected to vaccination).*

**Hypothesis 4a–b (H4a–b).**  *Exposure to COVID-19 vaccine information disseminated by online authority sources will be (a) positively related to hope (connected to vaccination) and (b) negatively related to fear (connected to vaccination).*

### 1.5. Health Beliefs, Fear, and Hope

According to Nabi [53,54], discrete emotions could direct cognitive resources to information that is expected to relieve negative emotions and reinforce positive emotions. Nabi et al. [55] considered negative cognitions to be enhancing one’s threat perception toward the risk to generate fear. As each discrete emotion can potentially generate a distinctive effect on activating adaptive behaviors, emotions elicited by cognitive appraisals can in turn result in behavioral responses relevant to the conditions of benefit or harm [56,57].

For instance, Champion et al. [58] argued that fear resulted from perceptions of threat implied a conceptual connection between fear and perceived barriers (also see [59]. Past studies have also shown that cognitive beliefs of a vaccine may engender discrete emotions, which may drive individuals to further seek risk information [60,61]. By implication, perceived barriers that evolve around psychological obstacles to COVID-19 vaccination (e.g., the vaccines could cause deadly side effects, pain) can be directly linked to fear toward vaccination. To test this theoretical proposition, a hypothesis is postulated below.

**Hypothesis 5 (H5).**  *Perceived vaccination barriers will be positively related to fear (connected to vaccination).*

According to Lazarus [43], the feeling of hope originates when a stimulus offers a relief or a better outcome than the current, less favorable situation. In other words, hope springs, if individuals foresee a better future outcome that is congruent with their goals. By extension, if individuals believe that COVID-19 vaccines are beneficial, they will anticipate a positive outcome of vaccination (i.e., to be protected from the virus), which may elicit their emotion of hope. Wang [62] found that while perceived benefits of receiving COVID-19 vaccination predicted vaccine uptake intent, the former also predicted the emotion of hopeful feelings toward vaccination, which further led to vaccination intent. As this conceptual link has only been tested in a very limited scope thus far [62], the following hypothesis is proposed to validate this presumptive theoretical connection:

**Hypothesis 6 (H6).**  *Perceived vaccination benefits will be positively related to hope (connected to vaccination).*

### 1.6. Information Exposure, Beliefs, Emotions and Information Engagement

Existing research on risk communication has suggested a positive relationship between risk information seeking and risk information engagement [63]. For instance, exposure to social media COVID-19 information was found to be positively related to engagement with the COVID-19 information on these online venues [17,18]. Yet, empirical research that examines audience engagement with COVID-19-related information on information source types (online authority vs. non-authority) has been lacking. Hence, the current study proposed two research questions to explore the linkages between exposure to online authority vs. online non-authority sources and vaccine information engagement.

**Research Question 1a–b (RQ1a–b).**  *Will (a) exposure to COVID-19 vaccine information disseminated by online non-authority sources and (b) exposure to COVID-19 vaccine information disseminated by online authority sources predict vaccine information engagement?*

Even though emotions like fear [64,65] and hope [66,67] have been found to link to vaccination uptake, their association with vaccine-related information engagement has yet to be explored. In the risk information context, most research has focused on information seeking behaviors, reporting their relationships with fear that was aroused by risk perceptions [61,68,69,70].

Empirical evidence on information engagement behaviors in relation to emotions has been limited. High-arousal emotions such as fear can trigger social transmission actions such as the dissemination of information in the public sphere [71,72]. Preliminary research has suggested that negative emotional responses triggered by fake news (non-pandemic and non-vaccination related) could enhance information engagement activities [73]. In the risk communication context, Han et al. [74] found that anger, instead of fear, toward the COVID-19 pandemic favorably predicted misinformation sharing. Tsang et al. [75] found that anger was associated with sharing of information from non-government/non-expert sources, whereas anxiety was related to sharing of information from government/expert sources during the COVID-19 pandemic. Anxiety was also named as a reason for sharing health-related misinformation or unverified health-related information [76,77] during the pandemic. Yet, the implications of fear as a discrete emotion connected to vaccination on vaccine information engagement are unknown.

Prior research has addressed the implications of positive emotions on risk information seeking but very little on *risk information engagement* behaviors. For example, initial research has demonstrated that hope connected to vaccination was found to favorably influence information seeking for COVID-19 vaccines [60,61,78]. As hope stems from optimistic expectancy for the future that can remedy the unsatisfactory status quo [55], individuals are likely to commit to active coping behaviors that can help achieve the desirable outcome when they feel hopeful [62]. Hence, hope could increase the action tendency to build thought-action repertoires for coping with the risk in relation to utilizing the information beneficial for developing coping strategies [13], with information engagement being one of such actions.

Few studies have investigated the relations between emotions (e.g., anxiety, anger) and *health information engagement* [74] and no research thus far has examined such relations particularly in the vaccination context. Likewise, even though empirical studies have validated the relations between emotions, vaccine misinformation, and information seeking, such relationships have yet to be examined in the information engagement context. The current study conceptualizes information engagement as a “response” (R) variable in the S-O-R framework and proposes the following hypotheses to extend the existing literature by verifying the conceptual relations between emotions and vaccine information engagement.

**Hypothesis 7 (H7a–b).**  *(a) Fear connected to vaccination and (b) hope connected to vaccination will be positively related to vaccine information engagement.*

Furthermore, no research has investigated how beliefs about vaccination benefits and barriers are linked to information engagement behavior. Therefore, how cognitive “organisms” (O) reflected in health beliefs may be associated with vaccine information engagement (R) is unknown. Due to the lack of empirical findings on such relationships, two research questions are raised below:

**Research Question 2a–b (RQ2a–b).**  *Will (a) perceived vaccination benefits and (b) perceived vaccination barriers predict vaccine information engagement?*

**Figure 1:**
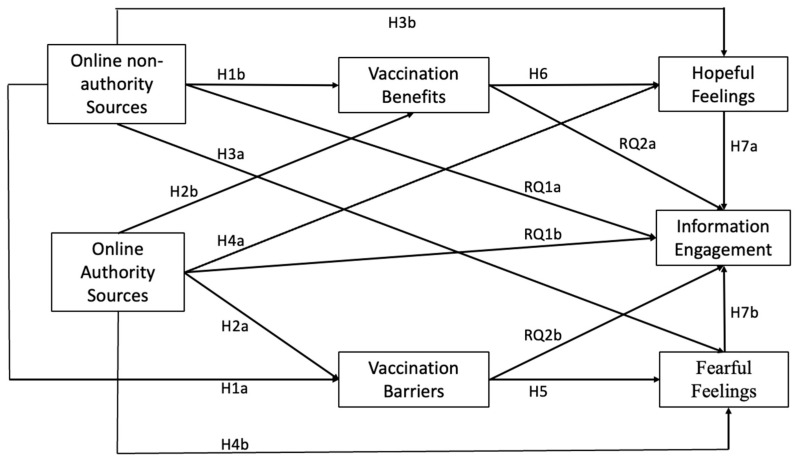
Proposed Conceptual Model.

To summarize the complex interrelationships between the theoretical constructs, a conceptual framework is shown below to illustrate them in conjunction with the proposed hypotheses and research questions (Figure 1). Specifically, both exposure to online non-authority and online authority sources (S or stimulus) are connected to cognitive and affective reactions toward vaccination (O or organism). The cognitive reactions of perceived vaccination benefits and perceived vaccination barriers (O) are, respectively, linked to the affective reactions of hopeful feelings and fearful feelings (O) connected to vaccination. Both cognitive and affective organisms (O) are then linked to vaccination information engagement (R or response).

## 2. Materials and Methods

### 2.1. Method

A survey was conducted with a national sample of the U.S. adult population. This sample was recruited by a U.S. survey service (Qualtrics). Data collection was completed during the week of 21 June 2021, when COVID-19 remained one of the leading causes for deaths in the U.S. The sample was selected using the following quotas: (1) equal gender split between males and females; and (2) three-way age group split: 18–30, 31–40, and 41 years and older.

### 2.2. Measures

Exposure to online non-authority sources was evaluated by adapting previous measures [26,79] that asked whether participants depended on different sources to learn about COVID-19 vaccines. The measures included three sets of online media venues excluding any type of authority (e.g., governments, health agencies, medical professionals, research scientists) on these venues: (1) social media (e.g., Facebook, Twitter, YouTube), (2) online communities/BBS (e.g., Reddit, Quora), and (3) search engine (e.g., Google, Bing). These items were combined to create a new variable of “exposure to online non-authority sources” (α = 0.85, M = 3.02, SD = 1.17).

Exposure to online authority sources was described by a single item reflecting online accounts for government offices or agencies (e.g., federal government, state government, CDC, local health agencies, and the like) and widely recognized non-government sources with authority status (e.g., John Hopkins University’s medical school, health professionals, medical researchers, and the like) (M = 3.64, SD = 1.04).

Fearful and Hopeful feelings associated with vaccination were each measured with three items adapted from Nabi and Prestin [55] on a five-point scale (ranging from 1 = none to 5 = very strong). These items asked participants to indicate how much they experienced different feelings associated with COVID-19 vaccines. Fear was reflected by the afraid, anxious and worried feelings; hope was illustrated by the hopeful, optimistic, and enthusiastic feelings. The confirmatory factor analysis confirmed the two discrete emotions: hope (α = 0.90, M = 3.16, SD = 1.30) and fear (α = 0.91, M = 2.47, SD = 1.25).

Perceived vaccination benefits were assessed by five items on a Likert scale (1 = strongly disagree and 5 = strongly agree), adopted from Sherman et al. [80] and Hornik et al. [81]. Sample items include (1) “If I get a COVID-19 vaccination, the chances of getting infected will go down significantly”. and (2) “If I get a COVID-19 vaccination, the chances of developing complications will go down significantly”. (α = 0.91, M = 3.62, SD = 1.03).

Perceived vaccination barriers were gauged with seven items on the same Likert scale, adopted from Lin et al. [82] and Forster et al. [83]. Example items include (1) “I worry that I would suffer bad side effects from the vaccines”; (2) “ I expect that COVID-19 vaccination will be very painful” and (3) “ I have concern about the efficacy of the COVID-19 vaccines”. (α = 0.91, M = 2.80, SD = 1.06).

Information engagement was measured by asking participants when they received information about COVID-19 vaccines, how often they performed the following behaviors: “posting/reposting it online”, “commenting the content online”, “liking” the content online”, and “discussing this with family and friends” (α = 0.88, M = 2.28, SD = 0.92). These measurement items, reported on a 4-point scale (from 1 = never to 4 = always), were adapted from Strekalova and Krieger [84].

Demographic characteristics included age, gender, race/ethnicity, annual household income and education level.

### 2.3. Statistical Analyses

The data analysis was conducted with the following statistical procedures, using the SPSS 28 program package. First, Cronbach’s inter-item reliability test was adopted to generate the measurement reliability coefficients (i.e., alpha values) for each theoretical construct measure. Second, descriptive statistics, including frequencies, means and standard deviations were computed for the demographic variables and all theoretical constructs. Third, a zero-order correlations analysis was applied to present the intercorrelations among the theoretical constructs. Fourth, the analysis of variance (ANOVA) procedure was implemented to compare the survey responses to the theoretical constructs between different age groups. Next, the SPSS AMOS 28 program package was utilized to conduct a confirmatory factor analysis to ascertain the measurement validity. This was followed by a path analysis that tested the research hypotheses and research questions illustrated in the proposed conceptual model.

## 3. Results

### 3.1. Descriptive Results

Data collected from the original sample contained 744 responses. Of these, 630 valid responses were retained after data cleaning and validation (e.g., removing cases with incomplete responses). The average age of the final sample was 39.9 years old (SD = 17.03), with the following racial/ethnic composition: 66.7% Caucasians, 15.1% African Americans, 8.3% Hispanic/Latino Americans, 5.4% Asian Americans, and 4.6% “other” categories. The median household income of the sample was USD 50,000–59,999 and the median education level reflected a 2-year college degree.

Table 1 shows the means, standard deviations and bivariate correlations of all variables. All variables were significantly correlated with each other except for the correlations between the following variable pairs: (1) exposure to authority sources and fear, (2) exposure to authority sources and perceived vaccination barriers, (3) fear and perceived vaccination benefits, and (4) hope and perceived vaccination barriers.

The confirmatory factor analysis for all the measurements yielded a good model fit, χ^2^ = 846.29, CMIN/df = 2.80, *p* < 0.001, CFI = 0.95, NFI = 0.93, IFI = 0.95, TLI = 0.95, and RMSEA = 0.05. All factor loadings were above 0.60.

### 3.2. Hypotheses Testing

**Figure 2:**
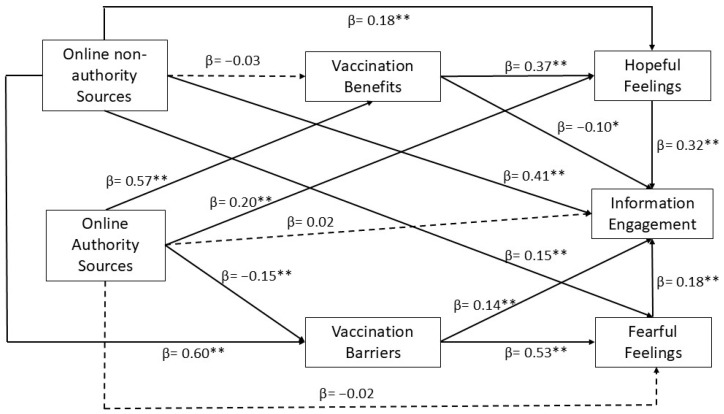
Original Path Analysis Results. ** *p* < 0.001; * *p* < 0.01.

The path analysis conducted to test the conceptual model retained a good fit, χ^2^ = 4.24, CMIN/df = 2.12, *p* = 0.12, CFI = 0.99, NFI = 0.99, IFI = 0.99, TLI = 0.99, RMSEA = 0.04 (Figure 2).

H1a-b postulate that exposure to online non-authority sources will be positively related to perceived vaccination barriers and negatively related to perceived vaccination benefits, sequentially. Results showed that exposure to online non-authority sources positively predicted perceived vaccination barriers (β = 0.60, *p* < 0.001) but not perceived vaccination benefits (β = −0.03, *p* = 0.43). Hence, H1a was validated but not H1b.

H2a-b assume that exposure to online authority sources will be negatively related to perceived vaccination barriers and positively related to perceived vaccination benefits in that order. Results demonstrated that exposure to online authority sources negatively predicted perceived vaccination barriers (β = −0.15, *p* < 0.001) and positively predicted perceived vaccination benefits (β = 0.57, *p* < 0.001). Both H2a and H2b were therefore supported.

H3a and H3b presume that exposure to online non-authority sources will be positively related to fear and negatively related to hope toward vaccination, respectively. Results exhibited that exposure to online non-authority sources was a positive predictor for both fear (β = 0.15, *p* < 0.001) and hope (β = 0.18, *p* < 0.001). Both H3a and H3b were thus validated.

H4a–b posit that exposure to online authority sources will be positively related to hope and negatively related to fear toward vaccination. Exposure to online authority sources did appear as a positive predictor for hope (β = 0.20, *p* < 0.001) but not fear (β = −0.02, *p* = 0.57). Hence, H4a was validated but not H4b.

H5 and H6 propose that perceived vaccination barriers and benefits will be positively related to fear and hope toward the vaccine, in that order. Results confirmed both H5 (β = 0.53, *p* < 0.001) and H6 (β = 0.37, *p* < 0.001). H7a and H7b maintain that fear and hope toward COVID-19 vaccines will be positively related to vaccine information engagement. Results showed that both fear (β = 0.18, *p* < 0.001) and hope (β = 0.32, *p* < 0.001) predicted vaccine information engagement, supporting H7a and H7b.

For RQ1a and RQ1b, results suggested that exposure to COVID-19 vaccine information disseminated by online non-authority sources positively predicted vaccine information engagement (β = 0.41, *p* < 0.001), but not exposure to COVID-19 vaccine information disseminated by online authority sources (β = 0.02, *p* = 0.53). Turning to RQ2a and RQ2b, results demonstrated that perceived vaccination benefits negatively predicted vaccine information engagement (β = −0.10, *p* = 0.003). By contrast, perceived vaccination barriers positively predicted vaccine information engagement (β = 0.14, *p* < 0.001).

**Figure 3:**
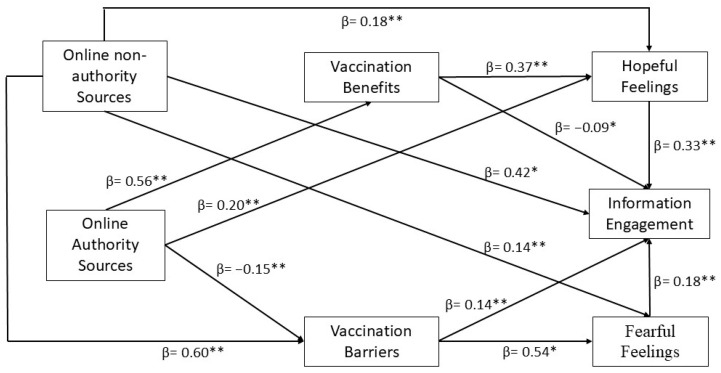
Modified Path Analysis Results. ** *p* < 0.001; * *p* < 0.01.

Based on the path analysis results, the three non-significant paths affiliated with H1b, RQ1b, and H4b were removed from the originally proposed conceptual model to present a modified conceptual model in Figure 3. The model showed that cognitive appraisals of the COVID-19 vaccination resulting from exposure to online authority vs. online non-authority sources are significantly related to emotions such as hope and fear (connected to the vaccines). Health beliefs and emotions are further linked to online users’ engagement with COVID-19 vaccine information.

Additional analyses were also conducted to test the differences in participant responses to the seven constructs between four different age groups. These four different age groups reflect the following age segments: 18–29 (Group 1), 30–39 (Group 2), 40–49 (Group 3), and 50 and older (Group 4). These age segments closely match the COVID-19 fatality statistics documented by the U.S. Centers for Disease Control and Prevention [85]. ANOVA results showed that significant differences were found in in all seven dependent variables between different age groups (Table 2).

Post hoc comparison results (Table 3) between age groups suggested that Group 1 (18–29), Group 2 (30–39) and Group 3 (40–49) all reported significantly higher exposure to non-authority sources than Group 4 (50 and older). For exposure to authority sources, Group 1 had significantly lower means than both Group 2 and Group 4, but not Group 3. As Group 1 perceived significantly lower vaccination benefits than the three older groups, Group 2 indicated significantly lower perceived vaccination benefits than the oldest Group 4. All three younger age groups reported significantly higher levels of perceived vaccination barriers than Group 4. While the only significant difference was detected between Group 2 and Group 3 for hopeful feelings, all three younger groups reported significantly stronger fearful feelings than Group 4. Finally, all three younger age groups demonstrated higher information engagement level than Group 4.

## 4. Discussion

The current study is among the first to validate a conceptual model that explores vaccine-related health beliefs and emotions in relation to information source exposure, as well as their roles in explaining vaccine information engagement. This study applied the S-O-R framework and the Health Belief Model to verify their validity in assessing vaccination-related information behaviors. Empirically, this study illuminates the psychological antecedents of vaccine information engagement—hope and fear connected to vaccination as well as perceived benefits and barriers of vaccination—as a result of exposure to both online non-authority and online authority sources.

Prior work has established that people spent a large amount of time viewing the information about important public health events through authority and non-authority sources [2,7]. The current study showed that exposure to online non-authority sources was associated with higher perceived barriers but unrelated to perceived benefits of vaccination. These findings align with the past literature showing that online information sources often disseminated misinformation about the vaccines [3], which led to vaccination hesitancy [32]. As misinformation casts doubt on or denies the effectiveness and safety of COVID-19 vaccines [33], exposure to online non-authority sources populated with such information could increase biased evaluations about the vaccines. It is worth noting that exposure to online non-authority sources was irrelevant to perceived benefits of vaccination. This result suggests that the information focusing on the harms instead of benefits of the vaccines might have been more effective, or the effects of these two types of information might have canceled each other out. The current study has thus extended the existing literature by proposing and validating a conceptual link from online non-authority media use to health beliefs about the COVID-19 vaccines.

Similarly, exposure to online authority sources positively predicted perceived vaccination benefits and negatively predicted perceived vaccination barriers. These results were congruent with prior research showing positive links between consumption of authority sources and knowledge about the pandemic [1,37]. By focusing on two health belief concepts, this study also helps to explain the favorable influences of exposure to authority sources (online or offline) on COVID-19 vaccine attitudes and adoption of prevention behaviors, as reflected in the recent literature [8,38,39,40].

Turning to the relationship between online authority sources with hope and fear, results indicated that accessing these sources was positively related to hope connected to vaccination but irrelevant to fear. These finding provided the first empirical evidence to illustrate the significant connection between accessing official sources for COVID-19 vaccine information and positive affective responses toward the vaccines. Yet, exposure to online authority sources had no influence on fearful feelings connected to vaccination. This finding may be indicative of how the information about the vaccination risks might have counterbalanced the information about vaccination benefits. Hence, online authority sources that promote vaccination as a prevention behavior may create a sense of hope for the public, without cultivating or reducing fear in them.

In contrast, the findings for the hypotheses addressing online non-authority sources indicated that these information sources had a significant relationship with fear connected to vaccination. The finding reported here is not surprising, considering the rampant dissemination of misinformation and disinformation about COVID vaccines in an infodemic environment [35]. This result is also consistent with existing research which demonstrated the negative emotional implications, including anxiety, depression, and reduced emotional well-being, that were associated with exposure to negative pandemic information disseminated in online media [49,50,51].

More interestingly, the current findings also confirmed that online non-authority sources had a significant relationship with hope as well. It is highly likely that the widely circulated inspirational personal stories about people receiving the vaccines with no concerns or side effects could elicit a sense of hope for the vaccines [86,87]. Considering that exposure to online non-authority sources failed to predict perceived vaccination benefits, it is interesting to see that exposure to such sources could enhance optimism emotion connected to vaccination [88]. Thus, accessing online vaccine information not distributed by authority sources may be associated with both stronger fear as well as greater hope connected to vaccination. As the transmission of misinformation and disinformation in the age of infodemic is unavoidable, dispelling the fear-inducing misbeliefs about vaccination remains a major risk communication challenge of the scientific community, policymakers, and public health agencies.

Findings also demonstrated that perceived vaccination barriers were positively related to fear connected to vaccination. While fear has been shown to grow out of threat appraisals of illnesses and health risks, the current study thus furthers the existing literature by exhibiting that fear toward a coping strategy (e.g., vaccination) could stem from negative appraisals of that strategy [55]. Study results also demonstrated that perceived benefits of vaccination had a positive association with hope connected to vaccination. This finding confirms how such positive health beliefs may help elicit hopeful feelings toward vaccination, consistent with limited past research [62]. Together, these results reported here validated the theoretical relationships between health beliefs for a risk-coping practice and discrete emotions [66].

Moreover, study findings showed that hope connected to vaccination was associated with increased engagement with vaccine information. This result expands past research which demonstrated that individuals with a hopeful feeling tended to take more proactive actions in response to a threat, including risk information seeking [60,61,78]. Similarly, a positive relation between fear connected to vaccination and vaccine information engagement was also revealed. This outcome resonates with past research suggesting that negative emotions could engender social behaviors to promote risk information sharing [74,75,76,77]. By implication, as perceived hope and fear connected to vaccination help facilitate vaccine information engagement, these findings also ascertain the role of messages that spread hope and fear in inducing high-arousal sentiments and behavioral responses during a pandemic [71,72].

Interestingly, study results revealed that exposure to online non-authority sources was positively related to vaccine information engagement. It is possible that inspirational stories about vaccines saving lives [89], conspiratorial stories about vaccines harming vaccinated individuals [90] and/or stories ambivalent about vaccine effectiveness [91] could motivate information engagement behavior such as likes, dislikes, comments, questions, and more. Of note, the current findings are the first empirical evidence addressing the relationship between exposure to online non-authority COVID-19 sources and information engagement.

By contrast, exposure to online authority sources only had an indirect effect on vaccine information engagement (through hopeful feelings) but no direct effect. A plausible explanation could be that exposure to authority information sources might have facilitated perceived information sufficiency [92], which promotes satisfaction with the vaccine information received and makes further information seeking or engagement unnecessary. An additional reason could be that the vaccine information conveyed through online authority sources tends to convey scientific facts, advisories, and policy announcements, which typically reflects a one-way communication approach without lending itself to stimulate information engagement behavior per se.

Furthermore, perceived vaccination barriers were also found to be positively associated with vaccine information engagement. Since exposure to non-authority sources significantly predicted perceived vaccination barriers and vaccine information engagement, it is plausible that those who reported perceived barriers to COVID-19 vaccination may also have a stronger motivation for information engagement—to express concerns to alert others or to express their own negative thoughts toward such adverse information—by verbally interacting with others [20,93]. Greater perceived vaccination benefits were also associated with lower vaccination information engagement activities. By implication, those who were convinced by or more satisfied with the information they have received about the vaccines were not compelled to engage in further information behavior to change their beliefs. In essence, a confirmation bias [94] might have negated the needs for such information engagement behavior.

In sum, the current study found differential effects of exposure to online authority and online non-authority vaccine information (S) on eliciting vaccination-related cognitions and affects (O), and information engagement (R) with one exception: eliciting positive emotion toward vaccination. On one hand, exposure to online authority sources demonstrated a direct negative effect on vaccination-barrier beliefs—and a direct positive effect on vaccination-benefit beliefs and hopeful feelings—without a direct effect on vaccine information engagement. On the other hand, exposure to online non-authority sources illustrated a direct positive effect on vaccination-barrier beliefs as well as hopeful and fearful feelings, aside from a heightened vaccine information engagement behavior. Hence, scientists and public health practitioners should consistently utilize all online and offline media outlets to present science-based risk communication to counter the damaging effects of vaccine misinformation and anti-vaccination rhetoric on public health from non-authority sources.

### 4.1. Practical Implications

This study offers several insights for government officials and public health practitioners that promote COVID-19 vaccines. First, as online non-authority sources were important channels for influencing public perceptions of the vaccines, it is essential for government authority sources to (1) advise the public to avoid accessing or believing information that has not been scientifically validated; (2) share the rationale behind evidence-based public policy approach; and (3) take responsibilities for any errors that have occurred in the information shared or the policy implemented. In essence, building and maintaining public trust in authority/expert sources is essential for communicating about vaccine risks and benefits as well as how vaccination is the best protective measure against COVID-19 transmission.

Second, it would be important to ensure the public that the only objective of the government health agencies—as well as the credentialed scientists and health professionals consulted to develop COVID-19 prevention guidance and policies—is to protect and advance public health in the U.S. Third, it would be beneficial to acknowledge the all-encompassing infodemic that has created confusion and misapprehension about the vaccines to curb the spread of false information and reduce fear. This can be done, for example, by identifying examples of false narratives, misleading images, and Deepfake videos, in addition to suggesting useful online search tips to help meet the public’ information seeking and engagement needs.

Fourth, risk communication professionals who construct the messaging strategy for COVID-10 vaccines should consider utilizing different content strategies and appeals when disseminating scientific information about the vaccines. For instance, it would be beneficial to tailor the vaccine messages to different age groups. As demonstrated in the current study, age can make a difference in how individuals respond to the vaccine information sources and content. Likewise, it would also be productive to promote these messages on different online venues that can best reach different audience segments to help solicit public feedback and information engagement.

Finally, this study was conducted with a sample recruited from the U.S. only. The practical implications for other countries may involve the consideration of unique cultural factors and different local political contexts, assuming that the public policy on vaccination recommendation and/or mandate are relatively similar. When it comes to the influence of cultural factors on COVID-19 vaccination behavior, the empirical evidence is mixed in the current infodemic environment. In particular, even though distinct cultural values (e.g., Hofstede’s cultural dimensions) were found to impact COVID-19 prevention behaviors [95], other empirical evidence indicated that cultural factors were not a valid or reliable explanation for a micro-level issue such as a pandemic and vaccine-related behavior [96]. A key finding in the COVID-19 literature suggests that trust in authority information sources such as governments and government health agencies remains the primary motivator for vaccination uptake [8,40,41].

### 4.2. Limitations

The current study has several limitations. First, the survey methodology adopted for this study relied on retrospective, self-reported data, which did not lend to causal attributions. An experimental study design could further validate the interrelations among the key variables. Second, this study focused on information sources, cognitive beliefs and affective responses regarding the vaccines, as well as their relations to information engagement behaviors. Future studies could incorporate additional constructs such as political leaning and normative influence to further explicate harmful information behaviors in an infodemic environment. Third, as this study focused only on information engagement behavior involving general online media and authority sources, it excluded other information sources such as traditional media outlets (e.g., popular cable news outlets and podcasts) and interpersonal sources (e.g., friends, family members, and faith communities) that can also influence information behavior [2]. Lastly, investigating the interaction effects on risk information engagement behavior resulting from accessing different information sources (e.g., scientists, governments, health care provider, family and friends) and information dissemination modalities/platforms (e.g., TV, radio, social media, online news, and interpersonal channels) could also produce additional explanations to the phenomenon under study.

## 5. Conclusions

Drawing from the S-O-R framework and the Health Belief Model, this study empirically contrasted how exposure to online authority vs. online non-authority information sources may be related to relevant cognitive and emotional factors to influence vaccination information engagement. The proposed conceptual model in the current study thus contributes to our understanding of how cognitive appraisals of and affective responses to risk information disseminated by different types of information sources may be related to risk information engagement behavior in a public health crisis.

In an infodemic environment, misinformation and disinformation could create biased beliefs about the COVID-19 vaccines to trigger emotional responses that can impede an individual’s vaccination uptake and contribute to vaccine hesitancy. Community-based education programs, health care providers, as well as friends and families, could all take action to share vaccine effectiveness information to counter vaccine misinformation and reduce vaccination barriers [97]. Future health communication campaigns should consider utilizing positive emotional appeals to elevate hope and lessen fear to prevent risk avoidance behavior. These campaigns should also be mindful of potential psychological reactance, e.g., [98,99], toward the risk information disseminated by government agencies and health professionals to motivate positive information engagement behavior to help promote the pro-vaccination messages.

## Figures and Tables

**Table 1:** Descriptives and Bivariate Correlations.

	1	2	3	4	5	6	7
1. Non-Authority Source Exposure							
2. Authority Source Exposure	0.37 **						
3. Information Engagement	0.67 **	0.30 **					
4. Fear Connected to Vaccination	0.44 **	0.08	0.50 **				
5. Hope Connected to Vaccination	0.32 **	0.48 **	0.44 **	0.14 **			
6. Perceived Vaccination Benefits	0.18 **	0.56 **	0.13 *	−0.04	0.52 **		
7. Perceived Vaccination Barriers	0.54 **	0.07	0.50 **	0.61 **	0.02	−0.11 **	
M	3.02	3.64	2.28	2.47	3.16	3.62	2.80
SD	1.17	1.04	0.92	1.25	1.30	1.03	1.06

** Correlation is significant at *p* < 0.01 (2 tailed). * Correlation is significant at *p* < 0.05 (2 tailed).

**Table 2:** ANOVA Results for Age Groups.

Dependent Variables	df (between-Group)	F	*p*
Exposure to Non-Authority Sources	3	29.18	***
Exposure to Authority Sources	3	4.13	0.007
Perceived Vaccination Benefits	3	16.20	***
Perceived Vaccination Barriers	3	23.61	***
Fear Connected to Vaccination	3	19.30	***
Hope Connected to Vaccination	3	2.66	0.048
Information Engagement	3	30.88	***

*** *p* < 0.001.

**Table 3:** Age Group Pairwise Comparisons.

	Group 1		Group 2		Group 3		Group 4		
	18–29 N = 176		30–39 N = 212		40–49 N = 83		50 and up N = 156		Group Comparisons *
	*M*	*SD*	*M*	*SD*	*M*	*SD*	*M*	*SD*	
Exposure to Non-Authority Sources	3.16	0.98	3.19	1.16	3.53	1.16	2.33	1.10	G1 > G4; G2 > G4; G3 > G4
Exposure to Authority Sources	3.41	1.10	3.73	1.03	3.70	1.04	3.74	0.95	G1 < G2; G1 < G4
Perceived Vaccination Benefits	3.27	0.89	3.60	1.09	3.75	.93	4.01	0.99	G1 < G2; G1 < G3; G1 < G4; G2 < G4
Perceived Vaccination Barriers	2.98	0.88	2.90	1.10	3.21	1.06	2.23	0.98	G1 > G4; G2 > G4; G3 > G4
Fear Connected to Vaccination	2.81	1.13	2.50	1.28	2.78	1.33	1.88	1.09	G1 > G4; G2 > G4; G3 > G4
Hope Connected to Vaccination	3.08	1.15	3.06	1.37	3.50	1.26	3.21	1.39	G2 < G3
Information Engagement	2.54	0.78	2.35	0.97	2.56	0.95	1.72	0.72	G1 > G4; G2 > G4; G3 > G4

* All comparisons are significant at *p* < 0.05.

## Data Availability

The data are available upon request.
